# A Four-Year Prospective Pilot Study of Newborn Screening for Late-Onset Proximal Urea-Cycle Disorders in Hyogo Prefecture in Japan

**DOI:** 10.3390/ijns12020039

**Published:** 2026-06-04

**Authors:** Tomoko Lee, Miki Matsui, Yoko Yokoyama, Ryosuke Bo, Hiroyuki Awano, Dai Kataoka, Masaaki Ueda, Toshinori Minato, Hironori Kobayashi, Yuki Hasegawa, Kei Murayama, Yasuhiro Takeshima

**Affiliations:** 1 Department of Pediatrics, Hyogo Medical University, Nishinomiya 663-8501, Japan; mi-matsui@hyo-med.ac.jp (M.M.); yoko1935963@yahoo.co.jp (Y.Y.); ytake@hyo-med.ac.jp (Y.T.); 2 Department of Pediatrics, Kobe University Graduate School of Medicine, Kobe 650-0017, Japan; ryobo@med.kobe-u.ac.jp; 3 Organization for Research Initiative and Promotion, Tottori University, Yonago 683-8503, Japan; awano@tottori-u.ac.jp; 4 Department of Pediatrics, Toyooka Hospital, Toyooka 668-8501, Japan; dai-kataoka@toyookahp-kumiai.or.jp (D.K.); m-ueda@ueda-babykids.jp (M.U.); toshinori-minato@toyookahp-kumiai.or.jp (T.M.); 5 Laboratories Division, Shimane University Hospital, Izumo 693-8501, Japan; bakki@med.shimane-u.ac.jp (H.K.); yukirin0329@yahoo.co.jp (Y.H.); 6 Department of Pediatrics, Matsue Red Cross Hospital, Matsue 690-8506, Japan; 7 Department of Pediatrics, Juntendo University, Tokyo 113-8421, Japan; kmuraya@mri.biglobe.ne.jp; 8 Department of Diagnostics and Therapeutics of Intractable Diseases, Intractable Disease Research Center, Graduate School of Medicine, Juntendo University, Tokyo 113-8421, Japan

**Keywords:** proximal urea-cycle disorders, hypocitrullinemia, hyperammonemia, newborn screening, citrulline, ornithine transcarbamylase deficiency, carbamoyl phosphate synthase 1 deficiency, N-acetylglutamate synthase deficiency, inborn errors of metabolism, mitochondrial disease

## Abstract

Proximal urea-cycle disorders (PUCDs), including N-acetylglutamate synthase deficiency (NAGSD), ornithine transcarbamylase deficiency (OTCD), and carbamoyl phosphate synthase 1 deficiency (CPS1D), cause hyperammonemia and impair neurological outcomes. Early detection of late-onset forms allows presymptomatic intervention to prevent hyperammonemia; however, reliable newborn screening (NBS) markers are lacking. This prospective pilot study in Hyogo Prefecture, Japan, evaluated hypocitrullinemia as a screening marker for late-onset PUCDs. Newborns with citrulline levels below the 0.05th percentile on NBS between June 2020 and May 2024 were enrolled in the study. Confirmatory diagnosis of PUCDs was performed using plasma amino acids, urinary organic acids, and genetic testing. During the first period (101,172 newborns), 11 newborns exhibited hypocitrullinemia; 10 underwent further evaluation. One newborn was diagnosed with CPS1D (compound heterozygous *CPS1* variants); another was later diagnosed with Leigh syndrome. The remaining eight cases were false positives, often associated with prematurity, poor feeding, or gastrointestinal disorders. A second dried blood spot (DBS) card protocol was introduced in the second period (34,694 newborns), reducing false positives. One neonatal-onset OTCD case was detected, and citrulline levels were normalized in six of the seven other cases. In summary, hypocitrullinemia can identify presymptomatic PUCDs, and requesting a second DBS card reduces false positives, supporting its feasibility for incorporation into NBS programs.

## 1. Introduction

The urea cycle is a metabolic pathway that detoxifies ammonia by converting it to urea and comprises six enzymes and two transporters [1]. Proximal urea-cycle disorders (PUCDs), including N-acetylglutamate synthase deficiency (NAGSD, OMIM #237310), carbamoyl phosphate synthetase 1 deficiency (CPS1D, OMIM #237300), and ornithine transcarbamylase deficiency (OTCD, OMIM #311250), result in hyperammonemia that can lead to severe neurological injury and death. The clinical phenotype ranges from neonatal onset to later onset, depending on residual enzyme activity. Neonatal-onset PUCDs, characterized by complete enzyme deficiency, typically present with severe hyperammonemia within the first few days of life, whereas late-onset PUCDs with partial enzyme deficiency manifest after the neonatal period. Late-onset PUCD may present with hyperammonemia at any age, often triggered by protein loading or increased catabolism. Although generally milder than the neonatal-onset form, late-onset PUCDs can still result in significant neurological sequelae and mortality [2,3].

PUCDs are treatable conditions. Early management with a protein-restricted diet and ammonia-scavenging medications, such as sodium benzoate and sodium phenylbutyrate, along with arginine or citrulline supplementation, can prevent hyperammonemia and improve prognosis, as recommended by international consensus guidelines [1]. In particular, for late-onset PUCDs, a window of opportunity often exists before the first hyperammonemic episode, underscoring the importance of early diagnosis and timely intervention.

Newborn screening (NBS) is an established public health strategy for the early detection and treatment of inherited metabolic disorders. However, PUCDs are not yet widely included in universal NBS programs globally, although certain regions, such as several states in the USA, have already incorporated them into their screening panels. This limited global adoption is largely because no universally accepted, highly reliable screening markers have been firmly established [1,4]. Although several candidate markers have been investigated, none have been widely accepted as sufficiently sensitive and specific for PUCD detection [5]. To improve screening accuracy, supplementary strategies such as measuring orotic acid levels in DBS have also been investigated [6].

Citrulline is a urea-cycle amino acid synthesized primarily in the mitochondrial matrix of enterocytes in the small intestine [7]. In Japan, citrulline has been routinely measured since the introduction of tandem mass spectrometry-based NBS in 2014, which currently screens for 20 primary disorders encompassing amino acid, organic acid, and fatty acid oxidation disorders [8]. In current NBS programs, elevated citrulline levels are used to screen for argininosuccinic aciduria (OMIM #207900), citrullinemia type 1 (OMIM #215700), and citrin deficiency (OMIM #603471). Although low citrulline levels (hypocitrullinemia) have been proposed as a potential screening marker for PUCDs, previous studies suggest that citrulline alone lacks sufficient sensitivity and reliability [5,9,10]. Moreover, prospective population-based data evaluating low citrulline levels in NBS are scarce, and retrospective analyses of NBS citrulline concentrations in patients with late-onset PUCDs remain limited.

In this context, our previous retrospective study demonstrated that patients with late-onset OTCD had significantly lower citrulline levels in presymptomatic NBS samples [11]. Although challenges related to sensitivity and specificity persist, these findings suggest that hypocitrullinemia may serve as a potential screening marker for PUCDs. To further investigate this hypothesis, we conducted a large-scale prospective pilot study in Hyogo Prefecture, Japan. This study focused on hypocitrullinemia in NBS to identify factors influencing citrulline concentrations in NBS and to evaluate its feasibility as a screening marker for PUCDs.

## 2. Materials and Methods

### 2.1. Study Design

In Hyogo Prefecture, the universal NBS system is organized through a coordinated network of perinatal medical institutions, screening laboratories, and local governments. When a screen-positive result is identified, the infants are referred to designated core medical centers for a definitive diagnostic workup. Specifically, for inherited metabolic disorders, these evaluations are primarily managed by core hospitals in the prefecture, chiefly the two major university hospitals: Hyogo Medical University Hospital and Kobe University Hospital.

This prospective pilot study was seamlessly integrated into this established regional framework to evaluate low citrulline levels as a potential screening marker for PUCDs. Because citrulline is already routinely measured in the conventional NBS program, no additional blood sampling or modification of assay procedures was required. Under this collaborative infrastructure, newborns identified with hypocitrullinemia on NBS underwent further diagnostic investigations, strictly following the existing clinical pipeline of Hyogo Prefecture.

#### Subjects

Newborns who underwent NBS in Hyogo Prefecture between June 2020 and May 2024 were eligible for inclusion in this two-phase study.

During the first study period (June 2020 to April 2023), all newborns with hypocitrullinemia detected in the initial NBS sample (first dried blood spot [DBS]) were directly referred for further evaluation to determine the underlying cause.

During the second study period (May 2023 to May 2024), a modified algorithm was implemented based on findings from the first phase. A new sample test with a second DBS was performed when factors potentially associated with hypocitrullinemia—such as prematurity, poor feeding, or intestinal disease—were present. If citrulline levels remained below the cut-off on the second DBS test, further diagnostic investigations were conducted. Newborns without these factors were referred directly for confirmatory testing.

### 2.2. MS/MS Analysis of Citrulline in NBS

Blood samples were collected as DBS on filter paper on postnatal days 4 or 5 for NBS. In Hyogo Prefecture, the DBS samples were analyzed at one of two laboratories, depending on the place of birth: the LSI Medicine laboratory (Kobe Medical Association) or JAPAN MEDICAL LABORATORY Co., Ltd. (Osaka, Japan).

In accordance with the primary target panels for universal NBS in Japan, five primary amino acids—phenylalanine, valine, leucine, methionine, and citrulline—are routinely measured and subject to external quality control at both laboratories.

Citrulline levels were measured using flow injection analysis-tandem mass spectrometry, with minor analytical differences between the laboratories. At the LSI Medicine Laboratory, citrulline was quantified using the MS^2^ Screening Neo II kit (Siemens Healthcare Diagnostics K.K., Tokyo, Japan), a commercially available non-derivatized amino acid and acylcarnitine MS/MS screening kit used in Japan, in conjunction with an API 3200 LC/MS/MS system (AB Sciex, Framingham, MA, USA). At JAPAN MEDICAL LABORATORY Co., Ltd., measurements were performed using a non-derivatized MS/MS method with an Acquity TQD system equipped with a 2777C Sample Manager and a 1525μ Binary HPLC Pump (Waters Corporation; Nihon Waters K.K., Osaka, Japan), together with the Labeled Amino Acid Standards Set A (NSK-A1-OP-1; Cambridge Isotope Laboratories, Inc., Tewksbury, MA, USA) and Neo SMAAT^®^ Compact (SEKISUI MEDICAL Co., Ltd., Tokyo, Japan), which is officially approved for evaluating multiple amino acids, including citrulline.

#### Citrulline Cut-Off Values

The cut-off value for DBS citrulline levels in NBS was defined as the 0.05th percentile of the screening population. This threshold was determined based on citrulline distribution histograms from the screening cohort and data from our previously reported retrospective study of patients with late-onset OTCD [11]. The 0.05th percentile was calculated separately for each laboratory, resulting in cut-off values of <4.0 μmol/L at the LSI Medicine laboratory and <5.0 μmol/L at the JAPAN MEDICAL LABORATORY Co., Ltd.

### 2.3. Confirmatory Testing

Confirmatory evaluation was performed for newborns identified with hypocitrullinemia. The assessment included a review of perinatal and family history, evaluation of clinical symptoms, and physical examination. Blood ammonia concentrations were measured, and plasma amino acid analysis was performed using an automated amino acid analyzer via standard clinical laboratory methods. Urinary organic acid analysis was conducted at the Rare Disease Comprehensive Treatment Center, Shimane University Hospital, using gas chromatography–mass spectrometry.

Genetic analyses of the *OTC*, *CPS1*, and *NAGS* genes were performed to establish a definitive diagnosis of PUCDs, including in cases without overt clinical or biochemical abnormalities, because late-onset PUCD could not be excluded. Genomic DNA was extracted from peripheral blood, and molecular genetic testing was performed using next-generation sequencing at the Kazusa DNA Research Institute. In one case (Case 7), only the *OTC* gene could be analyzed.

Specifically for Case 5, mitochondrial genome analysis was performed using genomic DNA extracted from blood and urine samples. Initial variant screening was conducted via next-generation sequencing (NGS), following the established protocol by Murayama et al. [12]. Subsequently, the mutation workload (heteroplasmy level) was precisely quantified using droplet digital PCR (ddPCR) to validate the NGS findings.

### 2.4. Ethical Approval

This study was approved by the institutional ethics committee of Hyogo Medical University (approval number: Rinhi-0414; approval date: 27 August 2019) and was conducted in accordance with the Declaration of Helsinki and relevant local regulations. Written informed consent was obtained from the parents or legal guardians of all participants.

## 3. Results

To identify background factors associated with low citrulline levels detected by NBS, all newborns with low citrulline concentration on the initial NBS (1st DBS) during the first study period (June 2020 to April 2023) underwent further evaluation, including clinical examination and confirmatory laboratory testing.

**Figure 1:**
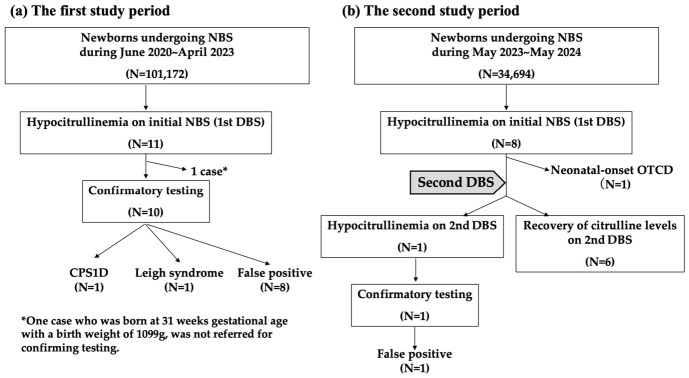
The screening process and results of the pilot study. NBS: newborn screening, DBS: dried blood spot; CPS1D: carbamoyl phosphate synthase 1 deficiency; OTCD: ornithine transcarbamylase deficiency.

Among 101,172 newborns screened during this period, 11 met the predefined criteria for low citrulline levels, and 10 underwent confirmatory evaluation (Figure 1a). One newborn (Case 6) was diagnosed with CPS1D, and another (Case 5) was subsequently diagnosed with Leigh syndrome. The remaining eight cases were classified as false positives. The clinical and biochemical characteristics of these 10 infants are summarized in Table 1.

Case 6 was a male newborn diagnosed with CPS1D. He was born at 37 weeks of gestation with a birth weight of 2582 g. The perinatal course was unremarkable, and there was no family history of urea-cycle disorders. The 1st DBS, obtained on day 5 of life, showed a markedly low citrulline concentration of 3.3 µmol/L (cut-off, <4.0 µmol/L). He was therefore referred to our hospital for further evaluation at 35 days of age. At presentation, he was clinically well with appropriate weight gain. The plasma ammonia concentration was 81 µmol/L. Plasma amino acid analysis demonstrated low citrulline (5.7 µmol/L; reference range, 17.1–42.6 µmol/L) and arginine (37.3 µmol/L; reference range, 53.6–133.6 µmol/L), accompanied by elevated glutamine (969.8 μmol/L; reference range, 422.1–703.8 µmol/L). Ornithine was within the normal range at 92.6 µmol/L (reference range, 31.3–104.7 µmol/L).

Urinary organic acid analysis showed no orotic acid. Although the patient was asymptomatic, these biochemical findings were highly suggestive of a PUCD. Subsequent genetic analysis identified no pathogenic variants in the *NAGS* or *OTC* genes but detected two compound heterozygous variants in the *CPS1* gene: c.1409A > G p. (Gln470Arg) and c.1913G > A p. (Arg638Gln). Both variants were previously unreported. According to the American College of Medical Genetics and Genomics/Association for Molecular Pathology guidelines, c.1409A > G was classified as a variant of uncertain significance (class 3; PM2, PP3), whereas c.1913G > A was classified as likely pathogenic (class 4; PM1, PM2, PM5, PP3). Based on these findings, a diagnosis of late-onset CPS1D was established. Oral arginine supplementation was initiated, and the patient has demonstrated normal growth and development without episodes of hyperammonemia to date.

Case 5 was a male infant born at 35 weeks of gestation with a birth weight of 2248 g. On the day of birth, he developed metabolic acidosis and transient arrhythmia, both of which improved with initial treatment. The 1st DBS obtained on day 4 of life showed a markedly low citrulline concentration (4.6 µmol/L; cut-off <5.0 µmol/L). He was referred to our hospital for further evaluation at 44 days of age. At presentation, he exhibited wheezing and reticular cyanosis; however, his overall condition was stable, and he had no vomiting or feeding difficulties. Blood ammonia levels were within the normal range, whereas plasma citrulline was below the detection limit. Genetic analyses of the *OTC*, *CPS1*, and *NAGS* genes identified no pathogenic variants, and PUCDs were therefore excluded. During follow-up, his clinical condition progressively deteriorated. Failure to thrive was noted at 4 months of age, followed by poor feeding, hypotonia, and developmental regression at 8 months. Blood analysis revealed elevated lactate (9.1 mmol/L) and pyruvate (0.32 mmol/L) concentrations. Brain magnetic resonance imaging performed at 8 months demonstrated bilateral symmetric basal ganglia lesions with lactate peaks on spectroscopy, findings highly suggestive of Leigh syndrome. Mitochondrial DNA analysis identified the *m.8993T > G* with a heteroplasmy rate of 98.2%, confirming the diagnosis of Leigh syndrome.

The remaining eight cases were classified as false positives after a comprehensive diagnostic evaluation. Four of these (Cases 1–4) were premature and had low birth weight. Poor feeding at the time of the initial NBS was documented in four cases (Cases 2–4 and 9), two of whom (Cases 3 and 4) also had gastrointestinal disorders. In these infants, plasma citrulline concentrations had normalized by the time of confirmatory testing, suggesting that prematurity, poor feeding, and gastrointestinal disturbances likely contributed to the initial hypocitrullinemia. Cases 8, 9, and 10 carried heterozygous variants in the *NAGS* and *CPS1* genes, respectively, suggesting that these genetic factors may be associated with their hypocitrullinemia. On confirmatory testing, plasma glutamine levels were 539.0, 718.8, and 633.0 µmol/L (reference range, 422.1–703.8 µmol/L), and arginine levels were 24.8, 69.5, and 96.9 µmol/L (reference range, 53.6–133.6 µmol/L), respectively.

These findings clarified the background factors underlying hypocitrullinemia detected by NBS and identified prematurity, low birth weight, poor feeding, and gastrointestinal disorders as major contributors to false-positive results. Based on these observations, a second study was conducted between May 2023 and May 2024. In this phase, a second DBS card protocol was applied to newborns who met any of the above criteria to reduce the incidence of false positives. Among 34,694 newborns screened during this period, eight met the criteria for low citrulline levels on the initial NBS (1st DBS) (Figure 1b). One newborn developed lethargy on day 4 of life, followed by impaired consciousness and hyperammonemia on day 5. The first DBS obtained on day 4 showed a citrulline concentration of 4.2 µmol/L (cut-off, <5.0 µmol/L), and the infant was subsequently diagnosed with neonatal-onset OTCD. The remaining seven newborns had at least one contributing factor—prematurity, low birth weight, or poor feeding—and therefore underwent a second DBS test. Six showed adequate recovery of citrulline levels (>10 µmol/L) on the 2nd DBS. One newborn with a cardiac tumor associated with tuberous sclerosis complex exhibited an initial DBS citrulline level of 3.5 µmol/L. Due to contributing factors of prematurity and poor feeding, a second DBS card was requested, which exhibited persistently low citrulline on the 2nd DBS (5.9 µmol/L). Although this value exceeded the cut-off (4.0 µmol/L), it remained within the borderline low range, warranting further evaluation. Comprehensive diagnostic assessment, including plasma amino acid analysis, urinary organic acid analysis, and genetic testing, revealed no abnormalities, and PUCDs were excluded.

When comparing the proportion of newborns requiring further examination, the first study showed a rate of 0.0099%. In contrast, the second period showed a rate of 0.0029%, demonstrating the effectiveness of the second DBS card protocol.

## 4. Discussion

This four-year prospective pilot study, involving over 135,000 newborns, clarifies the clinical factors associated with low citrulline levels (hypocitrullinemia) detected by NBS. One case of late-onset CPS1D was identified before symptom onset, demonstrating that citrulline can serve as a useful marker for presymptomatic detection of PUCDs. The screening algorithm applied—using citrulline as a first-tier marker on the initial DBS, followed by confirmatory biochemical and genetic testing—appears reasonable for detecting late-onset PUCDs. Importantly, because citrulline is already included in the current NBS panel, this strategy requires no additional analytes or modifications to existing laboratory infrastructure, allowing straightforward implementation within existing NBS systems.

Among PUCDs, OTCD is the most common, with an estimated incidence of approximately 1 in 80,000 live births, whereas CPS1D is considerably rarer, with prevalence estimates ranging from 1 in 539,000 to 1 in 975,000 globally, and approximately 1 in 800,000 in Japan [13,14,15,16]. In this context, the identification of one patient with neonatal-onset OTCD and one patient with late-onset CPS1D among 135,866 screened newborns in the present study was noteworthy. These findings suggest that undiagnosed or misdiagnosed individuals with PUCDs may exist in the general population, as late-onset forms often present with non-specific symptoms that result in delayed or incorrect diagnosis [17,18,19]. In contrast, as demonstrated in this study, presymptomatic diagnosis followed by appropriate intervention can prevent the development of hyperammonemia, underscoring the clinical significance of NBS-based diagnosis.

Previously reported challenges in citrulline-based screening for PUCDs include relatively high false-positive rates; however, the clinical factors underlying low citrulline levels have not been well characterized, as large-scale prospective data remain limited [9,13,14]. To clarify the causes of low citrulline values, including false-positive results, all newborns with low citrulline levels during the initial phase of this study were promptly referred for confirmatory testing. This approach revealed that prematurity, low birth weight, gastrointestinal disorders, and poor feeding were common causes of transient hypocitrullinemia, with citrulline levels normalizing on follow-up. Based on these findings, a second DBS card protocol was implemented in the subsequent phase of the study, substantially reducing false-positive results while preserving the detection of clinically significant cases.

In this study, cases without pathogenic variants in the *OTC*, *CPS1*, or *NAGS* genes were classified as false positives and were followed until at least 1 year of age. Notably, one patient who was later diagnosed with Leigh syndrome exhibited persistently low citrulline levels and harbored the m.8993T > G variant in *MT-ATP6*, which encodes a subunit of mitochondrial complex V. Previous reports have also described low citrulline levels in *MT-ATP6*-related mitochondrial disease, consistent with our observation suggesting that low citrulline may also serve as a potential biomarker for certain mitochondrial disorders, although further investigation is warranted [20,21,22]. No additional clinical features suggestive of mitochondrial disease were observed in the other cases; therefore, further mitochondrial genetic analyses were not performed. Cases 8, 9, and 10 carried heterozygous variants in *NAGS* or *CPS1*, which were considered to account for their low citrulline levels. These findings indicate that low citrulline levels on NBS may also reflect PUCD carriers or, in rare instances, mitochondrial diseases. With the recent expansion of clinical genetic testing, additional genetic analyses in newborns with low citrulline levels may help distinguish PUCDs from other underlying conditions.

The clinical spectrum of late-onset PUCDs is broad, and not all affected individuals exhibit low citrulline levels during the neonatal period [4,10,23]. Accordingly, this screening strategy is not designed to achieve complete sensitivity. Nevertheless, the early identification of relatively severe late-onset cases presenting in early infancy—before the development of hyperammonemic crises—has substantial clinical value. In our previous retrospective study, all patients with late-onset OTCD who developed symptoms between 1 and 2 years of age had exhibited low citrulline levels on NBS, suggesting that such cases might be detectable presymptomatically through citrulline-based screening [11].

Although a systemic follow-up for potential false negatives has not been fully established, no infants in Hyogo prefecture have been clinically diagnosed with PUCDs following the onset of symptoms during this four-year pilot study. While the possibility that patients present at a later age cannot be excluded, our findings suggest that this screening strategy is particularly useful for the early detection of relatively severe late-onset PUCDs manifesting in early childhood. In screening programs for disorders with heterogeneous phenotypes, minimizing false negatives is important; however, identifying even a subset of patients with more severe phenotypes before symptom onset can have a substantial clinical impact. This approach is consistent with the rationale underlying screening for several other inborn errors of metabolism and should likewise be considered in the context of PUCD screening.

Several alternative screening markers for PUCDs, including glutamine- or arginine-related ratios and the measurement of orotic acid, have been proposed [5,6,24,25,26]. However, glutamine is unstable in DBS, and neither glutamine nor orotic acid is routinely measured in the current Japanese NBS program, creating practical challenges for instrumentation and workflow. In contrast, the citrulline-based screening algorithm evaluated in this study requires no additional analytes and imposes no extra burden on newborns or screening laboratories. This simplicity represents a major advantage and supports the practical feasibility of implementation in real-world NBS settings. Although more advanced strategies may emerge in the future, this approach has the distinct benefit of being readily implementable within current screening systems.

Several limitations should be acknowledged. First, the citrulline cut-off value was provisionally set at approximately the 0.05th percentile, based on our previous retrospective analysis and the distribution of citrulline levels. To establish an optimal cut-off value, further large-scale prospective studies are needed, along with retrospective analyses of NBS citrulline levels in patients later diagnosed with PUCDs after symptom onset. Second, the follow-up duration was limited. Longer-term follow-up is required to fully assess screening sensitivity. Third, extended genetic evaluation was incomplete in false-positive cases. Comprehensive mitochondrial or broader metabolic genetic testing was not performed in all false-positive cases, and rare alternative etiologies may not have been fully excluded.

## 5. Conclusions

In this four-year prospective pilot study involving 135,866 newborns, the clinical factors associated with hypocitrullinemia detected by NBS were clarified. One patient with late-onset CPS1D was identified before symptom onset, suggesting that hypocitrullinemia can serve as a useful screening marker for PUCDs. The introduction of the second DBS card protocol was also shown to reduce false-positive results. These findings demonstrate that hypocitrullinemia can be feasibly incorporated as a screening marker for PUCDs within the existing NBS system.

## Figures and Tables

**Table 1:** Clinical and biochemical characteristics of newborns with hypocitrullinemia during the first study period.

Cases	Gestational Age (Weeks)	Birth Weight (g)	Sex	Perinatal Condition	Initial NBS (1st DBS)		Confirmatory Testing		Gene Variants in *OTC*, *CPS1*, and *NAGS*	Final Diagnosis
					Day	Citrulline (μmol/L)	Day	Plasma Citrulline (μmol/L)		
1	30	1566	M	Neonatal transient tachypnea	5	3.8	27	13.8	No	False positive
2	32	2162	M	Respiratory distress syndrome, Parenteral nutrition, Poor feeding	5	3.7	19	20.2	No	False positive
3	33	1840	M	21 trisomy, Duodenal atresia, Poor feeding	8	3.8	66	23.3	No	False positive
4	34	2350	F	Blood stool, Fasting, Poor feeding	5	4.8	23	22.0	No	False positive
5	35	2248	M	Metabolic acidosis, Transient arrhythmia	4	4.6	44	<LOD	No	Leigh syndrome
6	37	2582	M	No remarkable event	5	3.3	35	5.7	*CPS1*: c.1409A > G, c.1913G > A	CPS1D
7 *	38	2640	F	No remarkable event	5	3.5	27	8.5	No	False positive
8	39	2688	M	Neonatal jaundice	4	2.4	13	5.4	*NAGS*: c.504_505delinsTT (heterozygous)	False positive (NAGSD carrier s/o)
9	40	3374	M	Severe neonatal asphyxia, Poor feeding	5	4.8	43	6.3	*CPS1*: c.2798del (heterozygous)	False positive (CPS1D carrier s/o)
10	41	3106	M	No remarkable event	4	3.4	39	5.5	*CPS1*: c.302T > C (heterozygous)	False positive (CPS1D carrier s/o)

M: male; F: female; NBS: newborn screening; DBS: dried blood spot; LOD: limit of detection; NAGSD: N-acetylglutamate synthase deficiency; CPS1D: carbamoyl phosphate synthase 1 deficiency. * Only the *OTC* gene was analyzed in Case 7.

## Data Availability

The data presented in this study are not publicly available due to privacy and ethical restrictions related to patient information.

## Abbreviations

The following abbreviations are used in this manuscript:

NBS	Newborn screening
PUCDs	Proximal urea cycle disorders
NAGSD	N-acetylglutamate synthase deficiency
OTCD	Ornithine transcarbamylase deficiency
CPS1D	Carbamoyl phosphate synthase 1 deficiency
NAGS	N-acetylglutamate synthase
OTC	Ornithine transcarbamylase
CPS1	Carbamoyl phosphate synthase 1
DBS	Dried blood spot
